# Landscape Heterogeneity–Biodiversity Relationship: Effect of Range Size

**DOI:** 10.1371/journal.pone.0093359

**Published:** 2014-03-27

**Authors:** Naoki Katayama, Tatsuya Amano, Shoji Naoe, Takehisa Yamakita, Isamu Komatsu, Shin-ichi Takagawa, Naoto Sato, Mutsuyuki Ueta, Tadashi Miyashita

**Affiliations:** 1 Biodiversity Division, National Institute for Agro-Environmental Sciences, Tsukuba-shi, Ibaraki, Japan; 2 Conservation Science Group, Department of Zoology, University of Cambridge, Cambridge, United Kingdom; 3 Laboratory of Biodiversity Science, School of Agricultural and Life Sciences, University of Tokyo, Tokyo, Japan; 4 Japan Agency for Marine-Earth Science and Technology (JAMSTEC), Yokosuka, Kanagawa, Japan; 5 The Nature Conservation Society of Japan, Chuo-ku, Tokyo, Japan; 6 Biodiversity Center of Japan, Kamiyoshida, Fujiyoshida-shi, Yamanashi, Japan; 7 Japan Bird Research Association, Fuchu-shi, Tokyo, Japan; DOE Pacific Northwest National Laboratory, United States of America

## Abstract

The importance of landscape heterogeneity to biodiversity may depend on the size of the geographic range of species, which in turn can reflect species traits (such as habitat generalization) and the effects of historical and contemporary land covers. We used nationwide bird survey data from Japan, where heterogeneous landscapes predominate, to test the hypothesis that wide-ranging species are positively associated with landscape heterogeneity in terms of species richness and abundance, whereas narrow-ranging species are positively associated with landscape homogeneity in the form of either open or forest habitats. We used simultaneous autoregressive models to explore the effects of climate, evapotranspiration, and landscape heterogeneity on the richness and abundance of breeding land-bird species. The richness of wide-ranging species and the total species richness were highest in heterogeneous landscapes, where many wide-ranging species showed the highest abundance. In contrast, the richness of narrow-ranging species was not highest in heterogeneous landscapes; most of those species were abundant in either open or forest landscapes. Moreover, in open landscapes, narrow-ranging species increased their species richness with decreasing temperature. These results indicate that heterogeneous landscapes are associated with rich bird diversity but that most narrow-ranging species prefer homogeneous landscapes—particularly open habitats in colder regions, where grasslands have historically predominated. There is a need to reassess the generality of the heterogeneity-biodiversity relationship, with attention to the characteristics of species assemblages determined by environments at large spatiotemporal scales.

## Introduction

Landscape heterogeneity has long been considered a key determinant of biodiversity [1], [2]. Previous studies have reported contrasting associations between landscape heterogeneity and species richness, from positive to negative, and the negative effect is often reported to be the result of landscape fragmentation [1]–[5]. Metrics of landscape heterogeneity can also be regarded as good surrogates of species diversity because, in ecology, habitat diversity is associated with an increase in niche availability for species [2], [6]. Such association can also depend on spatial scales observed [7], [8]. To effectively manage heterogeneous landscapes to maintain biodiversity, we need to understand the mechanisms of such variability in the associations between landscape heterogeneity and species richness at multiple spatial scales [2], [7], [8].

One reason for such context-dependent patterns may be the difference among biomes in the sensitivity of species pools to fragmented landscapes, with lower sensitivity in the temperate zones of the Northern Hemisphere than in Oceania and tropical regions [9], [10]. Two mechanisms at different time scales can cause a varied response to landscapes. The first mechanism is evolutionary adaptation of species to landscape heterogeneity or homogeneity over long time scales [11], [12], and the second is selective extinction of species inhabiting homogeneous landscapes, such as forests or grasslands, by anthropogenic land-cover change over shorter time scales [10], [13]; the latter mechanism is called an “extinction filter” [14]. Because parts of the temperate regions have been subjected to anthropogenic disturbance for more than a thousand years, it is likely that the most sensitive species in these biomes have already become extinct [10], [15]. Therefore, consideration of the historical and contemporary land-cover patterns that may have determined regional species pools is important for predicting whether landscape heterogeneity has a positive or negative effect on species richness patterns.

It is important to note here that the spatial patterns of total species richness are based largely on those of wide-ranging species (i.e., species with large geographical range), because wide-ranging species usually contribute many more distribution records to species richness patterns than do narrow-ranging species [16]–[18]. In regions historically associated with landscape heterogeneity, species adapted to spatially and temporally variable environments, such as habitat generalists [19], [20] are expected to be widespread, whereas species adapted to homogeneous and stable environments may already have become extinct or may be distributed over a relatively narrow range. If this prediction is true, then any positive association between landscape heterogeneity and total species richness would be formed largely by the responses of wide-ranging species but not of narrow-ranging species of high conservation priority. However, this hypothesis has rarely been tested for species pool across terrestrial landscapes.

We addressed this hypothesis by using nationwide bird survey data from Japan. Land cover in Japan is mainly forest (66% of the total area), but farmland (12%) and grassland (<3%) have long been maintained by human activity, creating a mosaic landscape that has existed for thousands of years (often called *satoyama*; [21]). Such heterogeneous landscapes support a variety of plants, invertebrates, and vertebrates [22], [23]. In addition, historical land cover varies along a 1500-km latitudinal gradient within Japan: during the Last Glacial Maximum (LGM), when conditions were cooler and drier than now [24], grasslands could have been widespread, particularly in areas at high latitudes or altitudes (i.e., with cool and dry conditions) [25]. After the LGM, grassland has been maintained by human activities since more than thousand years ago, and its area had been at least 10% of the total area until early 20th century [25]. In accordance with the above-described current and historical land cover in Japan, we present here the following four predictions of the relationship between landscape heterogeneity and biodiversity. First, total species richness is positively associated with landscape heterogeneity, as reported in earlier studies [23]. Second, the richness and abundance of wide-ranging species, which consist mainly of habitat generalists, are positively associated with landscape heterogeneity. Third, the richness and abundance of narrow-ranging species, consisting mainly of habitat specialists, are, in contrast, highest in homogeneous landscapes, and the majority of narrow-ranging species are open-landscape specialists rather than forest specialists, given the smaller area of open habitats (i.e., grassland and farmland) than forests in Japan. Fourth, the richness of open-landscape specialists is particularly high in areas with low temperature, reflecting the high historical and current prevalence of grasslands there.

## Methods

### Ethics Statement

We obtained non-disclosure data on a nationwide survey of breeding birds in Japan from Ministry of the Environment as a part of “the Monitoring Sites 1000 Project” [26]. This survey did not require any approval for animal care and use because it was an observational field study, not involving the capture and handling of wild animals or their maintenance in captivity.

### Bird Data

The nationwide annual survey of breeding bird species in the “Monitoring Sites 1000” project has been conducted since 2004 [26], [27]. We used data between 2004 and 2009, which was available to us at the start of our study. The survey consists of over 300 1-km survey transects that are located in farmland, grassland, agriculture–forest mosaic, and forest across the country. While walking at 2 km h^−1^ along the transect line in the morning (between sunrise and 0900 h) during the breeding season (mid-May to early-July), skilled volunteers record all bird species observed or heard within 50 m of the line. Each transect was visited from less than once a year up to six times a year (from three to 24 times in total over the six years). But a survey year at each transect was not provided to us. We could only obtain the information for the number of surveyed transects in each year; more than 50 transects were surveyed in each year (range: 50–118 transects) and thus using only transects with the fixed number of survey years would considerably reduce sample size. Thus we used all the data for six years while accounting for differences in the numbers of surveys among transects in analysis (see below).

**Figure 1:**
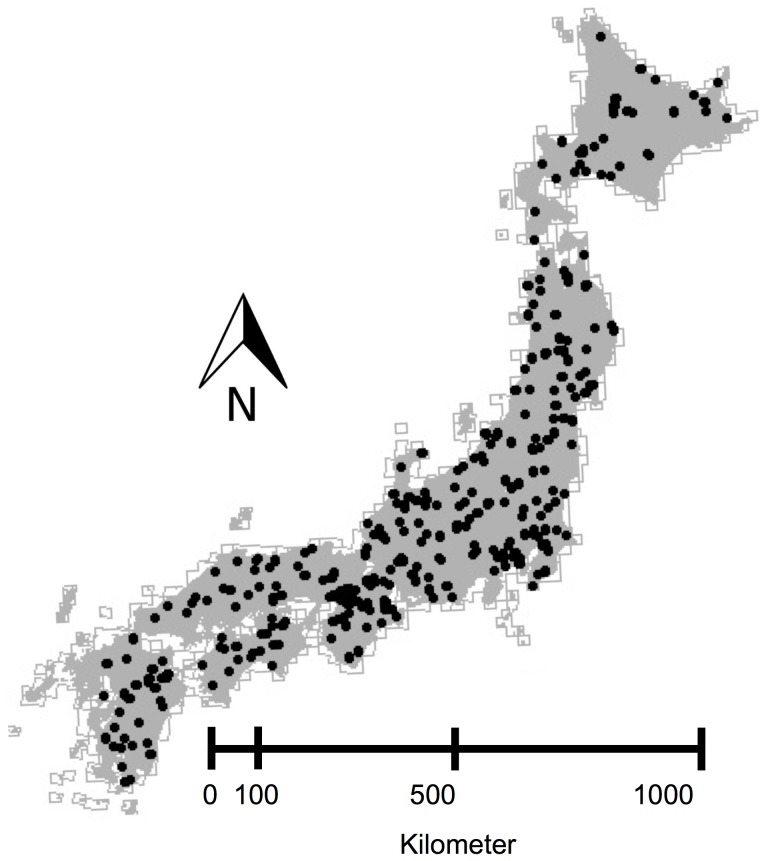
Locations of 313 study transects across the main four islands of Japan.

The bird data excluded transects (1) that were outside the four main islands of the Japanese Archipelago so as to control for island-size effects [28]; and (2) for which climate or habitat data could not be obtained. Consequently, we used records from 313 transects out of 363 for the following analyses (Figure 1). Although 177 species, including waterbirds, were recorded in the 313 transects between 2004 and 2009, we focused on 113 native terrestrial species that breed within Japan to investigate the effect of terrestrial landscape heterogeneity on bird species (see the full list of bird species in Appendix S1 in File S1).

Species range size in Japan was estimated based on the number of 20-km grid squares (over 1200 grid squares in total) in which each species was present, which was obtained from the sixth National Surveys on the Natural Environment from 1998 to 2002 [29] (range size is shown in Appendix S1 in File S1). These data are based on the bird atlas survey that aimed to assess the presence/absence of each species in all 20 km by 20 km grid cells in Japan while the survey for the “Monitoring Site 1000” project is conducted only at selected transects to investigate abundance and its changes. Thus we used the former to estimate species' range size. Using the range-size data, we defined wide-ranging species as those present in more than about one-third of the grid squares (≥400 grids) and the others (<400 grids) as narrow-ranging species (Appendix S2 in File S1). This yielded 38 wide-ranging and 69 narrow-ranging species; six raptor species were not grouped because their range data were not available. Results were qualitatively similar even when using 200 or 100 grids as a threshold (Appendix S3 in File S1).

In the analyses of data on the abundance of individual species, to avoid bias in our estimates of responses to landscape heterogeneity we used only 57 species that were observed in more than 20 transects. Consequently, we compared species-level responses to landscape heterogeneity between 38 wide-ranging and 19 narrow-ranging species.

### Climate and Energy

We obtained annual mean, minimum, and maximum temperature, and annual precipitation from Mesh Climate Value 2000 (available for purchase a CD-ROM at [30]). The data were 30-year (1971–2000) means of monthly and annual values within 1-km^2^ grids spread over a contiguous nationwide grid. We also obtained potential and actual evapotranspiration from the MeteoCrop DB (downloadable at [31]). For all climate and energy variables, we calculated mean values within 1 km from each transect line using ArcGIS ver. 9.3 [32].

### Topography and Land Cover

We obtained mean and range of elevation from Geospatial Information Authority of Japan (downloadable at [33]) and land-cover variables from 1∶50,000-scale vegetation maps based on the fifth Japanese National Survey of the Natural Environment (1994–1998) (available for a CD-ROM at [26]). Land-use patches larger than 0.25 ha can generally be identified on these maps. We aggregated all the legends of the vegetation maps in terrestrial habitats into the following eight land-cover types: (1) evergreen broad-leaved forest, (2) evergreen coniferous forest, (3) deciduous broad-leaved forest, (4) natural conifer plantation, (5) alpine vegetation, (6) grassland, (7) farmland, and (8) urban area. To investigate appropriate spatial scales at which habitat heterogeneity affected species, we calculated the proportion of each land cover within (1) 50 m (local scale) and (2) 1, 3, 5, and 10 km (landscape scales) from each transect using ArcGIS ver. 9.3. We then prepared three variables that represent landscape heterogeneity. First, Simpson's diversity index was calculated on the basis of the seven land-cover types (excluding urban area) at each of the four landscape scales. A higher value of Simpson's diversity index means greater compositional heterogeneity [2]. Second, edge density was calculated by dividing the sum of perimeter length of each land-cover patch (excluding urban area) by the total area of patches. A higher edge density means greater configurational heterogeneity [2]. Third, the proportion of forest cover was calculated by aggregating the first four land-cover types shown above. The proportion of forest cover showed a strong negative correlation with that of open habitat (i.e., sum of grassland and farmland; *r* = −0.85 to −0.82) at all landscape scales, reflecting that forest and open habitats constitute a large proportion (88% to 90% on average) of the total land cover of Japan. Thus the intermediate level of forest cover represents a heterogeneous landscape with mixtures of forest and open habitats, which has been reported as a key to support farmland biodiversity such as spiders and frogs in Japan [21], [34],[35]. We then investigated the relationship among the three landscape variables at the four landscape scales and found that Simpson's diversity index and edge density were highest with the intermediate proportion of forest cover at 1- and 3- km scales (Appendix S4 in File S1), and the two variables were moderately to highly correlated at all the four spatial scales (*r* = 0.55–0.65). We therefore considered Simpson's diversity index/edge density and the proportion of forest cover to be an index of fine- and coarse-grained landscape heterogeneity, respectively.

### Selection of Explanatory Variables

Each explanatory variable was standardized for statistical analyses. To test nonlinear effects, we adopted linear and quadratic terms for each variable (except for Simpson's Diversity Index and Edge density), yielding the following 13 variables in the analyses: number of surveys (Effort and Effort^2^), annual mean temperature (AMT and AMT^2^), annual precipitation (APP and APP^2^), actual evapotranspiration (AET and AET^2^), proportion of forest cover at the local (FRT_local_ and FRT_local_^2^) and landscape scales (FRT and FRT^2^) and Simpson's diversity index (SDI) or edge density (EGD). Effort and Effort^2^ were used only for the analyses of species richness. In the analyses of abundance, we used the number of individuals divided by the number of surveys as the response variable. To avoid multicollinearity, we did not use the other variables, which were moderately to highly correlated with the 13 selected variables (|*r*|>0.6). Minimum and maximum temperatures were highly correlated with AMT (>0.92). Potential evapotranspiration was closely correlated with AET (>0.99). Although AET was also highly correlated with AMT (0.77), we did not exclude this variable because AET was the only variable that directly represented available energy. We did not use mean elevation due to a high correlation with the range of elevation (*r* = 0.67–0.83). The range of elevation was strongly correlated with FRT (0.72–0.76). Thus we compared the effects of FRT and the range of elevation in separate models, and found that FRT tended to be more important variable than the range of elevation based on AIC value (Appendix S5 in File S1), indicating the importance of FRT as a direct measure of landscape heterogeneity. The proportions of grassland and farmland were also not used owing to the negative correlations with FRT (see “Topography and land cover”). Although FRT_local_ was moderately correlated with FRT (*r* = 0.39–0.71), we did not exclude this variable to separate the effect of local-scale heterogeneity (behavioral responses such as foraging habitat selection) from that of landscape-scale heterogeneity (population size). SDI and EGD was also correlated (0.55–0.65), and thus we did not use these variables simultaneously in one model but examined the importance of each variable by comparing AIC values of two models in which only one of the two variables was added (Appendix S6 in File S1). Because the model with SDI performed better than the model with EGD, we showed only results of the former in the main text. Finally, we checked for multicollinearity among the 13 variables using the VIF. All of our selected variables had VIF<6.5, suggesting that multicollinearity was not a serious problem [36].

### Statistical Analysis

In the analyses, the response variables were total species richness, the species richness of each group (wide-ranging and narrow-ranging species), and the abundance (i.e. number of individuals per survey) of each species (57 species) at each transect. These response variables were log(*x*+0.5)-transformed to normalize their distributions. Ignoring the effect of autocorrelation in model residuals could inflate type I errors and decrease the precision of parameter estimation [37]. Thus, we first regressed the species richness of each group on the selected 13 explanatory variables using non-spatial generalized linear models with normal distribution and identity link. We constructed four models with 1-, 3-, 5-, and 10-km scales for landscape variables. We then generated Moran's I correlograms of the regression residuals using the ‘ncf’ package [38] in R [39]. For most of the models, a significant positive autocorrelation was found within 150 km.

We therefore used simultaneously autoregressive error models [40] and the ‘spdep’ package [41] in R. For each model, we explored the “best” neighborhood distance by comparing the values of the Akaike Information Criterion (AIC) [42] among five distance classes (10, 30, 50, 100, and 150 km), using row standardization techniques for neighbor weights. We then used the distance class with the lowest AIC value as the neighborhood distance of each model. For both species richness and the abundance of each species, the best distance varied from 10 km to 150 km, depending on the species group or species (results not shown).

After we had determined the neighborhood distance, we investigated the spatial scale at which landscape variables most strongly influenced species. We first used backward stepwise variable selection to find the best model (set of explanatory variables)—i.e. the one with the lowest AIC value—at each of the four landscape scales. We then compared the AIC values of the best models at the four landscape scales, as well as those of the null models, to determine the best spatial scale. We calculated ΔAIC (the difference between each model's AIC and AIC_min_, i.e. the AIC of the best model), and employed the following criteria to determine the performance of a particular model relative to that of the best model: ΔAIC<2, substantial support; ΔAIC<7, considerably less support; and ΔAIC<10, essentially no support [42]. In some cases, the ΔAIC values at several different scales were <2. However, because the estimated responses to landscape heterogeneity were qualitatively similar among the best spatial scales in such cases, we used the model with the lowest AIC value as the best model.

We then used the estimated regression coefficients in the best model to obtain a predicted response of each species richness and abundance to each explanatory variable. To classify the response to landscape heterogeneity, we used only the proportion of forest cover, not Simpson's diversity index, because the former was included in the best model in much more cases than the latter (see Results). We categorized the predicted response of each species to the proportion of forest cover as follows: (1) open-habitat species: predicted abundance peaks when the proportion of forest cover is <25%, (2) mosaic-habitat species: abundance peaks when the proportion of forest cover is 25–75%, (3) forest species: abundance peaks when the proportion of forest cover is >75%, and (4) no response: proportion of forest cover is not included in the best model.

Finally, we examined the importance of an interaction term between annual mean temperature and FRT by adding the term into the best model for each species group and comparing the AIC values. We particularly focused on this interaction term to test the fourth hypothesis; if the richness of open-landscape specialists is higher in cold areas than in warm ones, it may suggest the importance of high historical and current prevalence of grasslands in northern or high-altitude regions.

## Results

### Total Species Richness

**Figure 2:**
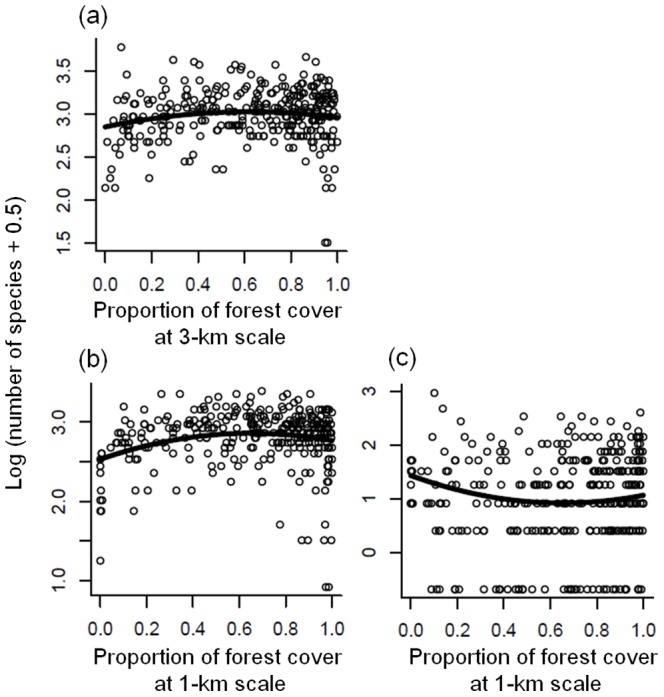
Relationship between species richness and proportion of forest cover at a landscape scale. (a) total species; (b) wide-ranging species; (c) narrow-ranging species. Regression lines are based on the estimated coefficients in the best simultaneous autoregressive model, using mean values other than the proportion of forest cover at the landscape scale.

For total species richness, the 3-km buffer was the best landscape scale (i.e. the one with the lowest AIC value), although the ΔAIC values for the other three scales were <2.5 (Table 1). The explanatory variables selected in these models were similar among spatial scales (Table 1), so the following result is based on the best model at the 3-km scale. The ΔAIC value of the null model was >100, indicating essentially no support for the null model. In the best model, linear or quadratic terms (or both) of survey effort, climate, energy, and land-cover variables were included (Table 1; Appendix S7 in File S1). For the proportion of forest cover at the landscape scale, the quadratic term was included and its coefficient was negative (Table 1), showing a hump-shaped response of total species richness to forest cover, with a peak at 62% cover (Figure 2a). Total species richness also showed a hump-shaped pattern with forest cover at the local scale, with the peak at 70% (Appendix S7 in File S1). Simpson's diversity index was not included in the best model.

**Table 1:** *Z* values (estimates/standard errors) of linear (L) and quadratic (Q) terms of each variable in the best simultaneous autoregressive models for total species richness and each species richness group at four landscape scales.

			Effort		AMT		APP		AET		FRT_local_		FRT		SDI
	AIC	Intercept	L	Q	L	Q	L	Q	L	Q	L	Q	L	Q	L
Total species															
1 km	15.2	67.8	4.5	−2.5		−8.1	−3.0			2.1	−1.5	−3.8	1.6	−1.6	
**3 km**	**14.9**	**73.8**	**4.4**	**−2.4**		**−7.8**	**−2.8**			**2.3**		**−5.5**		**−2.5**	
5 km	16.5	69.6	4.3	−2.2		−8.3	−3.5			2.2		−6.1	2.2		
10 km	17.2	74.1	4.5	−2.4		−7.7	−3.0			2.5		−6.2		−2.2	−1.7
Wide-ranging species															
**1 km**	**49.2**	**57.3**	**3.5**	**−2.2**	**4.3**	**−10.1**		**−3.5**		**1.8**	**−1.7**	**−4.4**		**−4.2**	
3 km	51.8	57.4	3.6	−2.3	4.3	−9.7		−3.4		2.1	−1.7	−5.7		−3.8	
5 km	58.9	53.0	3.4	−2.1	4.3	−10.1	−1.7	−2.2		2.0	−1.9	−6.4	3.1		2.0
10 km	63.1	60.3	3.4	−2.1	3.7	−9.8		−3.5		2.1	−1.6	−6.4		−1.7	
Narrow-ranging species															
**1 km**	**666.7**	**10.9**	**3.3**	**−1.5**	**−9.5**	**−2.1**				**2.0**				**3.1**	
3 km	669.9	10.2	4.8		−9.3	−2.2				1.6				2.7	
5 km	671.7	11.3	3.5	−1.8	−9.2	−1.8				2.0		2.1			
10 km	671.7	11.3	3.5	−1.8	−9.2	−1.8				2.0		2.1			

For each species richness, the landscape scale with the lowest AIC value is shown in bold. A blank space means that the variable is not included in the best model. Abbreviations: Effort: number of surveys, AMT: annual mean temperature, APP: annual precipitation, AET: actual evapotranspiration, FRT_local_: proportion of forest cover at a local (50-m) scale, FRT: proportion of forest cover at a landscape scale, SDI: Simpson's diversity index.

### Wide-Ranging vs. Narrow-Ranging Species

For the richness of wide-ranging species the 1-km buffer was the best landscape scale; the ΔAIC values for the other scales ranged from 2.6 to 13.9 (Table 1), suggesting considerable superiority of the model at the 1-km scale (i.e., the best model) relative to the models at the other scales. The ΔAIC value of the null model was >200, indicating no support for the null model. The set of explanatory variables and their coefficients in the best model were similar to those for total species richness (Table 1; Appendix S7 in File S1). For forest cover at the landscape scale, the quadratic term was included and its coefficient was negative (Table 1), showing a hump-shaped response to forest cover with the peak at 65% cover (Figure 2b). Wide-ranging species richness also showed a hump-shaped pattern at the local scale, with the peak at 63% cover (Appendix S3 in File S1). Simpson's diversity index was not included in the best model.

For the richness of narrow-ranging species, the 1-km buffer was the best landscape scale; the ΔAIC values for other scales ranged from 3.1 to 4.9 (Table 1), suggesting that the model at the 1-km scale was the single best model. The ΔAIC value of the null model was >80, indicating no support for the null model. For forest cover at the landscape scale, the quadratic term was included but its coefficient was positive (Table 1), resulting in a U-shaped response to forest cover with the peak at 0% cover, i.e., open habitats such as grassland and farmland (Figure 2c). Forest cover at the local scale and Simpson's diversity index were not included in the best model.

**Figure 3:**
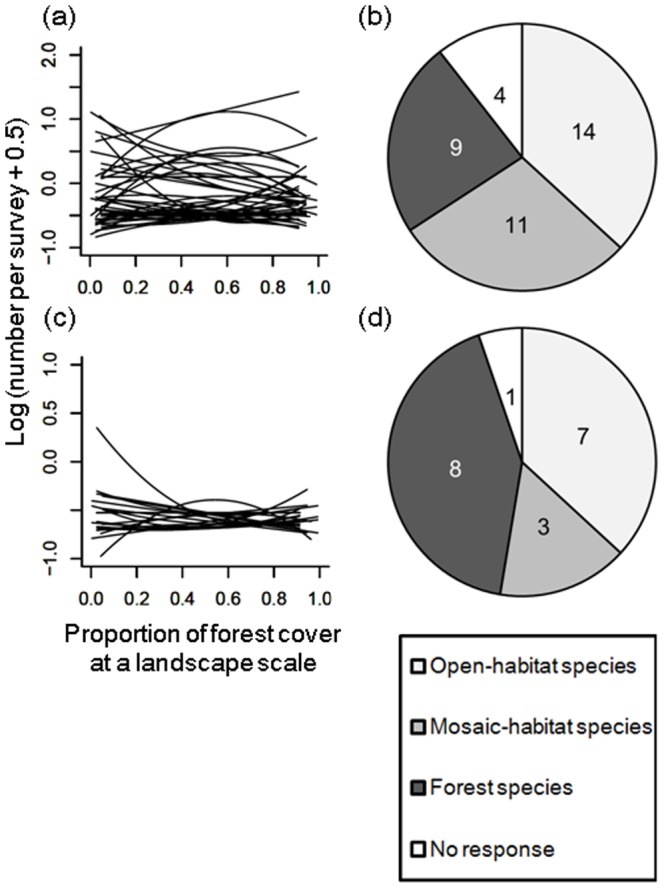
Response of each species to proportion of forest cover at a landscape scale. (a, c) Relationships between abundance of each species and proportion of forest cover at a landscape scale and (b, d) pie charts showing the proportions of species with the four categories of response type (open-habitat species, mosaic-habitat species, forest species, and no response) among (a, b) 38 wide-ranging species, and (c, d) 19 narrow-ranging species. Regression lines for each species were calculated in the same way as those for species richness (see explanation in the caption to Figure 2). Criteria for categorization of each species according to the proportion of forest cover are given in the text.

The species-level analysis (see Appendix S8 in File S1 for coefficients in the best model of each species) revealed that the three types of responses to landscape heterogeneity (open-habitat, mosaic-habitat, forest species) were well mixed in wide-ranging species (Figures 3a, 3b) and 28.9% of wide-ranging species were categorized as mosaic-habitat species, showing the highest abundance in heterogeneous landscapes (i.e., 25% to 75% forest cover) (Figure 3b). In contrast, among narrow-ranging species, the proportions of mosaic-habitat species were much lower (15.8%) than those of open-habitat and forest species (36.8% and 42.1%; Figures 3c, 3d).

### Interaction Terms

**Figure 4:**
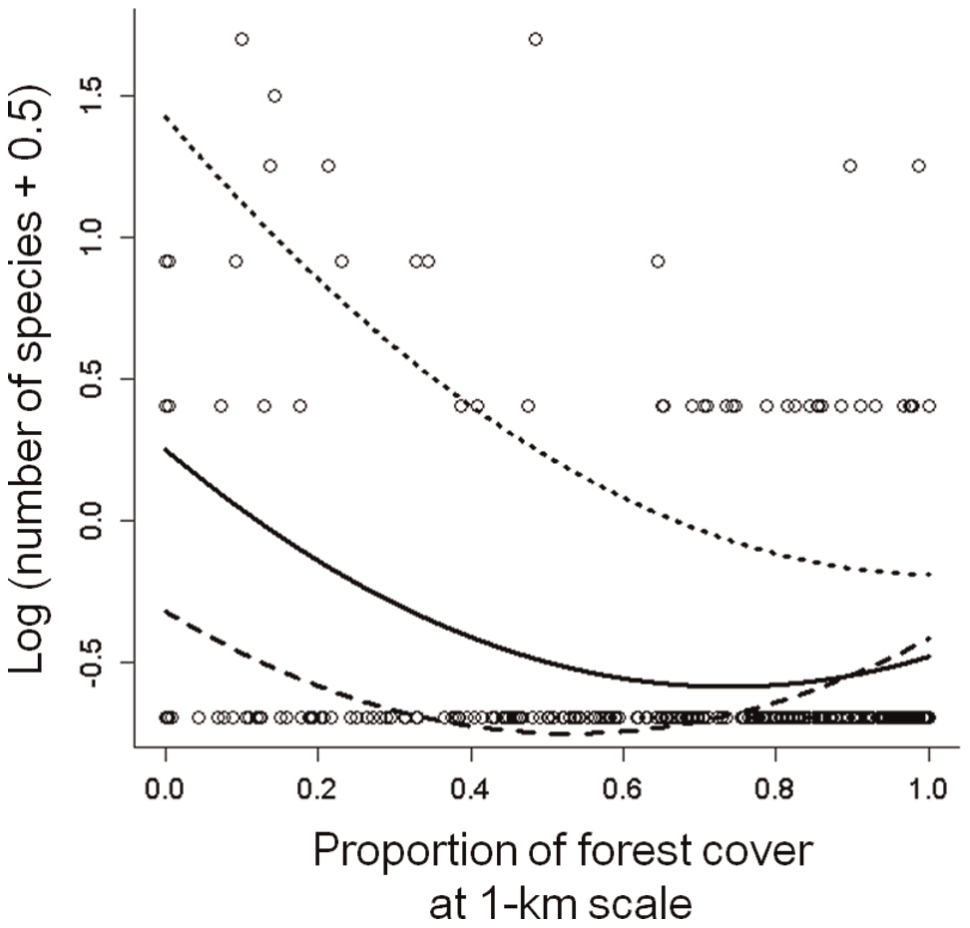
Effect of interaction between annual mean temperature and proportion of forest cover on the richness of narrow-ranging species. For narrow-ranging species, the definition of 100 grids is used (see Appendix S2 in File S1). Each point represents the relationship between the richness of narrow-ranging species and proportion of forest cover in each transect. Regression lines are based on the coefficients estimated with the simultaneous autoregressive model that incorporated the interaction term between these variables, at low temperature (upper 2.5% in the range of values in annual mean temperature, dotted line), mean temperature (50%, solid line), and high temperature (97.5%, broken line). Model performance is shown in Appendix S9 in File S1.

Adding the interaction term between annual mean temperature and the proportion of forest cover improved model performance (i.e. achieved a lower AIC value) only for narrow-ranging species richness (Appendix S9 in File S1); the negative effect of forest cover on narrow-ranging species richness was stronger in colder areas than in warmer ones (Figure 4), i.e., the richness of open-landscape specialists was higher in cold areas than in warm ones.

## Discussion

The results largely supported our original hypotheses. The total richness of terrestrial breeding bird species in Japan was highest in heterogeneous landscapes represented by an intermediate level of forest cover but not Simpson's diversity index and edge density (see also Appendix S6 in File S1). This suggests that the coarse-grained heterogeneity (agriculture-forest mosaics) was more important than the fine-grained heterogeneity for terrestrial birds in Japan, where agriculture-forest mosaics have been maintained for more than thousands of years. The abundance and richness of wide-ranging species were also highest in heterogeneous landscapes, whereas narrow-ranging species did not prefer heterogeneous landscapes in terms of either abundance or richness. Species-level analysis showed that for wide-ranging species, three types of responses to landscape heterogeneity (open-habitat, mosaic-habitat and forest species) were well mixed (Figures 3a, 3b). This indicates that the highest value of total species richness and richness of wide-ranging species in moderate forest cover resulted from both the positive effect of habitat heterogeneity *per se* and mass effect from source habitats (i.e., open-habitats and forest) to sink habitat (mosaic habitat). For narrow-ranging species, our result does not exclude the importance of heterogeneity within each landscape component, such as variations in vegetation type in grassland habitats. It will be useful to test the effect of local- and landscape-scale heterogeneity on biodiversity in future studies.

**Figure 5:**
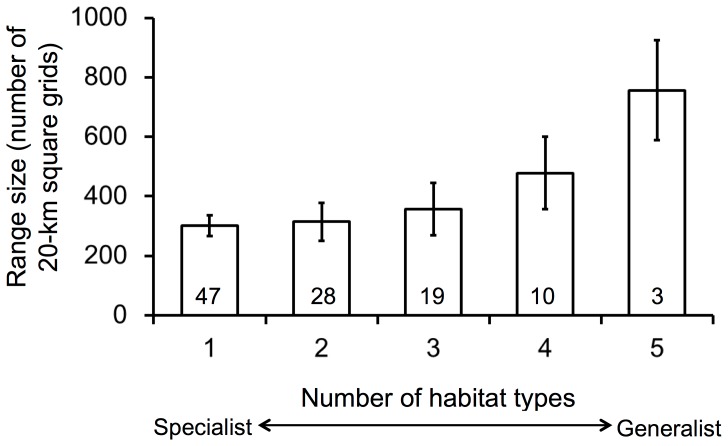
Relationship between number of habitat types used by a species and range size. For range size, the number of 20-km-square grids occupied by the species (obtained from [27]) is used for 107 out of 113 terrestrial bird species in Japan (except for six raptor species without range-size data). Values inside bars and error bars indicate sample size and standard error, respectively.

One possible explanation for the observed difference in responses to landscape heterogeneity between wide-ranging and narrow-ranging species is a difference in habitat breadth: generalist species tend to be wide-ranging, whereas specialist species tend to be narrow-ranging in Japan (Figure 5). In heterogeneous landscapes, habitat generalists can be widespread because they benefit from the supplementary or complementary use of multiple habitat types [43] and a reduction in the abundance of competitive dominant species, i.e., habitat specialists. In Japan, agricultural–forest mosaic landscapes have been historically common (see Introduction), explaining why these generalist species have flourished as wide-ranging species. However, the pattern in Figure 5 may also result from an artefactual relationship between niche breadth and distribution; niche breadths have often been determined for more individuals at a greater number of sites for widespread species than for restricted ones [44]. We could not perform rigorous statistical tests such as a randomization test (e.g., [45]) because only range-size data but not distribution pattern was available to us [29]. In future studies, such tests are needed to reveal the role of habitat niche breath as well as niche position on occurrence patterns in each species.

It is worth noting that the positive association between species richness of wide-ranging species and landscape heterogeneity was observed at both the local and the landscape scale. This suggests that landscape supplementation or complementation occurs at multiple spatial scales. There are two different processes that can cause such a pattern. First, different species may respond to heterogeneity at different spatial scales [34]. Second, each species can benefit from heterogeneity at multiple spatial scales. In our study, 9 out of 57 species exhibited a positive response to local-scale heterogeneity; 10 responded positively to landscape-scale heterogeneity and 4 to heterogeneity at both scales, giving a total of 23 such species (Appendix S8 in File S1). In either of the two processes, the existence of fractal-like landscapes, which exhibit repeated emergence of heterogeneous landscapes at different spatial scales, is likely to enhance the richness and abundance of these species; this may be the key to the coexistence of many species [46], [47].

In contrast to the case with wide-ranging species, the richness of narrow-ranging species was highest in open habitats with little forest cover, and the overall response to forest cover was U-shaped (Figure 2c). This implies that narrow-ranging species consist mainly of both open- and forest-landscape specialists, a finding that is also supported by the results of the species-level analysis (Figures 3c, 3d). The high proportions of open-landscape specialists among narrow-ranging species may be explained by the small area of their habitats, particularly grassland (<3% of the total area). Given that grassland was more widespread in Japan in from the LGM to early 20th century than it is today [25], it is possible that open-landscape specialists were historically more widespread but have suffered range contractions due to the loss, fragmentation, and degradation of their habitat. If this trend in land use continues in the future [21], [48], the range sizes of open-landscape specialists will further decline, further increasing the risk of local and regional extinctions. Our results also showed that the richness of open-landscape specialists was higher in cold areas than in warm ones (Figure 4). The grassland in cold regions has been maintained for a long time (i.e., since the LGM), and such temporal stability may have allowed more open-habitat species with a narrow distribution range to survive in these regions, enhancing the contribution of these species to the regional species pool there. But historical land-cover data has not been established yet in Japan and thus this explanation remains to be ascertained.

Partly contrary to our hypothesis, many forest specialists were also narrow-ranging species; this cannot be explained by the large size of Japan's forested area (66% of the total area). This indicates that the range size of forest species is determined by other factors such as the quality of habitats, climate, species interactions and dispersal barriers. In addition, reduction in areas of overwintering habitats may explain small range size of forest specialists; 28 out of 48 forest specialists breeding in Japan are summer visitors that overwinter in Southeast Asia (calculated from the work of [29]) and ongoing deforestation in these countries is a serious threat to the biodiversity of species, including birds [49], [50]. Earlier studies seem to support this explanation: migratory bird species have shown more severe range contractions than non-migratory species during the last few decades in Japan [51], [52]. More studies are clearly needed to separate out the effect of changes in breeding and overwintering habitats on the abundance and distribution of forest specialists in Japan.

Our results provide new insight into the heterogeneity–biodiversity relationship. To date, a large number of studies have reported a positive association between landscape heterogeneity and biodiversity, particularly in regions historically associated with landscape heterogeneity, such as parts of Europe, North America, and east Asia [1], [5], [22], [23], [53]. However, this might just represent the responses of wide-ranging species and ignore the responses of narrow-ranging species with high conservation priority, as shown by our study. Recent studies have also shown that threatened birds in farmland [54] or endemic soil fauna in mountainous areas [55] tend to be more negatively affected by landscape heterogeneity than are common species. We have further shown that our hypothesis is applicable to species in various terrestrial habitat types, including forest, grassland and farmland, by using comprehensive high-resolution distribution data covering terrestrial bird species and spanning different climatic regions. As spatial patterns in species richness do not necessarily correspond with those in phylogenetic and functional diversity [56], it will also be important to understand associations between these other biodiversity measures and landscape heterogeneity. Moreover, we have provided evidence that homogeneous open landscapes become the most important for narrow-ranging species in cold-climate regions, probably reflecting differences in species pools among temperate regions. More studies are needed in the future, including those of many more regions with different climatic and land-cover histories, because such studies would uncover the importance of both historical environments and contemporary human activities in determining the assemblages of species with varying preferences for heterogeneity. We believe that this is a challenging task that needs to be addressed urgently to determine appropriate conservation management strategies for different regions and biomes.

## Supporting Information

File S1 **Supporting Appendixes.**
(DOC)
