# Supplementary material for: Landscape Heterogeneity–Biodiversity Relationship: Effect of Range Size
Source: PLoS One. 2014 Mar 27;9(3):e93359. doi: 10.1371/journal.pone.0093359 (PMC3968173; doi:10.1371/journal.pone.0093359)
Supplement: File S1 — Supporting Appendixes. (DOC) [file pone.0093359.s001.doc]

**Appendix S1.** The full list of 113 terrestrial bird species in this study. Species’ range size in Japan was defined as the number of 20-km grid squares (over 1200 grid squares in total) in which each species was present, based on the sixth National Surveys on the Natural Environment (see main text). Rang size data is not available for six raptor species. For each species, abundance is shown by the percentile.

|  | Abundance (number per survey) | | | | | Observed at | Range |
| --- | --- | --- | --- | --- | --- | --- | --- |
| Scientific name | Min | 25 | 50 | 75 | Max | >20 sites | size |
| *Gorsachius goisagi* | 0.0 | 0.0 | 0.0 | 0.0 | 0.3 |  | 19 |
| *Pernis apivorus* | 0.0 | 0.0 | 0.0 | 0.0 | 0.2 |  |  |
| *Milvus Migrans* | 0.0 | 0.0 | 0.0 | 0.0 | 11.7 | Yes | 776 |
| *Haliaeetus albicilla* | 0.0 | 0.0 | 0.0 | 0.0 | 0.3 |  |  |
| *Accipiter gentilis* | 0.0 | 0.0 | 0.0 | 0.0 | 0.8 |  |  |
| *Accipiter gularis* | 0.0 | 0.0 | 0.0 | 0.0 | 0.2 |  | 61 |
| *Accipiter nisus* | 0.0 | 0.0 | 0.0 | 0.0 | 0.3 |  | 74 |
| *Buteo buteo* | 0.0 | 0.0 | 0.0 | 0.0 | 0.5 |  | 180 |
| *Butastur indicus* | 0.0 | 0.0 | 0.0 | 0.0 | 1.7 | Yes | 278 |
| *Spizaetus nipalensis* | 0.0 | 0.0 | 0.0 | 0.0 | 0.2 |  |  |
| *Circus spilonotus* | 0.0 | 0.0 | 0.0 | 0.0 | 0.3 |  |  |
| *Falco peregrinus* | 0.0 | 0.0 | 0.0 | 0.0 | 0.3 |  |  |
| *Falco tinnunculus* | 0.0 | 0.0 | 0.0 | 0.0 | 0.8 |  | 53 |
| *Tetrastes bonasia* | 0.0 | 0.0 | 0.0 | 0.0 | 0.7 |  | 47 |
| *Syrmaticus soemmerringii* | 0.0 | 0.0 | 0.0 | 0.0 | 0.7 | Yes | 192 |
| *Phasianus colchicus* | 0.0 | 0.0 | 0.0 | 0.0 | 4.0 | Yes | 495 |
| *Grus japonensis* | 0.0 | 0.0 | 0.0 | 0.0 | 0.2 |  | 24 |
| *Gallinago hardwickii* | 0.0 | 0.0 | 0.0 | 0.0 | 1.6 |  | 178 |
| *Streptopelia orientalis* | 0.0 | 0.0 | 0.5 | 1.1 | 8.0 | Yes | 1072 |
| *Sphenurus sieboldii* | 0.0 | 0.0 | 0.0 | 0.0 | 23.0 | Yes | 450 |
| *Cuculus fugax* | 0.0 | 0.0 | 0.0 | 0.0 | 0.8 | Yes | 236 |
| *Cuculus canorus* | 0.0 | 0.0 | 0.0 | 0.0 | 6.0 | Yes | 480 |
| *Cuculus saturatus* | 0.0 | 0.0 | 0.0 | 0.0 | 3.0 | Yes | 672 |
| *Cuculus poliocephalus* | 0.0 | 0.0 | 0.0 | 0.5 | 6.2 | Yes | 628 |
| *Otus lempiji* | 0.0 | 0.0 | 0.0 | 0.0 | 0.2 |  | 11 |
| *Ninox scutulata* | 0.0 | 0.0 | 0.0 | 0.0 | 0.3 |  | 132 |
| *Strix uralensis* | 0.0 | 0.0 | 0.0 | 0.0 | 0.3 |  | 182 |
| *Caprimulgus indicus* | 0.0 | 0.0 | 0.0 | 0.0 | 0.3 |  | 124 |
| *Hirundapus caudacutus* | 0.0 | 0.0 | 0.0 | 0.0 | 2.0 |  | 43 |
| *Apus pacificus* | 0.0 | 0.0 | 0.0 | 0.0 | 5.5 |  | 103 |
| *Halcyon coromanda* | 0.0 | 0.0 | 0.0 | 0.0 | 5.3 | Yes | 229 |
| *Jynx torquilla* | 0.0 | 0.0 | 0.0 | 0.0 | 0.8 |  | 48 |
| *Picus awokera* | 0.0 | 0.0 | 0.0 | 0.3 | 2.0 | Yes | 524 |
| *Picus canus* | 0.0 | 0.0 | 0.0 | 0.0 | 0.3 |  | 58 |
| *Dryocopus martius* | 0.0 | 0.0 | 0.0 | 0.0 | 0.2 |  | 51 |
| *Dendrocopos major* | 0.0 | 0.0 | 0.0 | 0.2 | 3.2 | Yes | 526 |
| *Dendrocopos leucotos* | 0.0 | 0.0 | 0.0 | 0.0 | 1.0 | Yes | 145 |
| *Dendrocopos minor* | 0.0 | 0.0 | 0.0 | 0.0 | 0.2 |  | 18 |
| *Dendrocopos kizuki* | 0.0 | 0.2 | 0.7 | 1.2 | 11.2 | Yes | 927 |
| *Pitta brachyura* | 0.0 | 0.0 | 0.0 | 0.0 | 1.0 |  | 29 |
| *Alauda arvensis* | 0.0 | 0.0 | 0.0 | 0.0 | 32.0 | Yes | 488 |
| *Riparia riparia* | 0.0 | 0.0 | 0.0 | 0.0 | 14.8 |  | 44 |
| *Hirundo rustica* | 0.0 | 0.0 | 0.0 | 1.3 | 18.2 | Yes | 713 |
| *Hirundo daurica* | 0.0 | 0.0 | 0.0 | 0.0 | 2.3 |  | 130 |
| *Delichon urbica* | 0.0 | 0.0 | 0.0 | 0.0 | 10.5 | Yes | 348 |
| *Motacilla cinerea* | 0.0 | 0.0 | 0.0 | 0.2 | 4.0 | Yes | 747 |
| *Motacilla alba* | 0.0 | 0.0 | 0.0 | 0.0 | 5.5 | Yes | 457 |
| *Motacilla grandis* | 0.0 | 0.0 | 0.0 | 0.0 | 2.7 | Yes | 478 |
| *Anthus hodgsoni* | 0.0 | 0.0 | 0.0 | 0.0 | 2.2 |  | 200 |
| *Pericrocotus divaricatus* | 0.0 | 0.0 | 0.0 | 0.0 | 4.8 | Yes | 224 |
| *Hypsipetes amaurotis* | 0.0 | 0.7 | 3.8 | 7.3 | 31.3 | Yes | 1104 |
| *Lanius tigrinus* | 0.0 | 0.0 | 0.0 | 0.0 | 0.2 |  | 10 |
| *Lanius bucephalus* | 0.0 | 0.0 | 0.0 | 0.0 | 7.7 | Yes | 668 |
| *Troglodytes troglodytes* | 0.0 | 0.0 | 0.0 | 0.0 | 8.0 | Yes | 432 |
| *Prunella rubida* | 0.0 | 0.0 | 0.0 | 0.0 | 0.5 |  | 29 |
| *Erithacus akahige* | 0.0 | 0.0 | 0.0 | 0.0 | 3.5 |  | 182 |
| *Luscinia calliope* | 0.0 | 0.0 | 0.0 | 0.0 | 4.2 |  | 79 |
| *Luscinia cyane* | 0.0 | 0.0 | 0.0 | 0.0 | 4.8 | Yes | 373 |
| *Tarsiger cyanurus* | 0.0 | 0.0 | 0.0 | 0.0 | 4.3 |  | 106 |
| *Saxicola torquata* | 0.0 | 0.0 | 0.0 | 0.0 | 7.8 |  | 165 |
| *Monticola solitarius* | 0.0 | 0.0 | 0.0 | 0.0 | 0.5 |  | 202 |
| *Zoothera dauma* | 0.0 | 0.0 | 0.0 | 0.0 | 1.2 | Yes | 363 |
| *Turdus sibirica* | 0.0 | 0.0 | 0.0 | 0.0 | 1.2 |  | 89 |
| *Turdus cardis* | 0.0 | 0.0 | 0.0 | 0.2 | 4.2 | Yes | 505 |
| *Turdus chrysolaus* | 0.0 | 0.0 | 0.0 | 0.0 | 2.5 | Yes | 352 |
| *Urosphena squameiceps* | 0.0 | 0.0 | 0.0 | 0.7 | 5.5 | Yes | 821 |
| *Cettia diphone* | 0.0 | 0.8 | 2.2 | 4.3 | 34.5 | Yes | 1155 |
| *Locustella pryeri* | 0.0 | 0.0 | 0.0 | 0.0 | 16.8 |  | 5 |
| *Locustella fasciolata* | 0.0 | 0.0 | 0.0 | 0.0 | 2.7 |  | 118 |
| *Locustella ochotensis* | 0.0 | 0.0 | 0.0 | 0.0 | 0.5 |  | 43 |
| *Locustella lanceolata* | 0.0 | 0.0 | 0.0 | 0.0 | 4.7 |  | 24 |
| *Acrocephalus bistrigiceps* | 0.0 | 0.0 | 0.0 | 0.0 | 36.0 | Yes | 152 |
| *Acrocephalus arundinaceus* | 0.0 | 0.0 | 0.0 | 0.0 | 26.5 | Yes | 353 |
| *Phylloscopus borealis* | 0.0 | 0.0 | 0.0 | 0.0 | 5.0 | Yes | 175 |
| *Phylloscopus tenellipes* | 0.0 | 0.0 | 0.0 | 0.0 | 4.7 |  | 218 |
| *Phylloscopus coronatus* | 0.0 | 0.0 | 0.0 | 0.2 | 13.7 | Yes | 586 |
| *Regulus regulus* | 0.0 | 0.0 | 0.0 | 0.0 | 4.0 |  | 105 |
| *Cisticola juncidis* | 0.0 | 0.0 | 0.0 | 0.0 | 44.0 | Yes | 258 |
| *Ficedula narcissina* | 0.0 | 0.0 | 0.5 | 1.7 | 10.7 | Yes | 757 |
| *Cyanoptila cyanomelana* | 0.0 | 0.0 | 0.2 | 0.8 | 5.7 | Yes | 780 |
| *Muscicapa dauurica* | 0.0 | 0.0 | 0.0 | 0.0 | 4.7 | Yes | 189 |
| *Terpsiphone atrocaudata* | 0.0 | 0.0 | 0.0 | 0.0 | 5.5 | Yes | 320 |
| *Aegithalos caudatus* | 0.0 | 0.0 | 0.2 | 1.2 | 28.3 | Yes | 711 |
| *Parus palustris* | 0.0 | 0.0 | 0.0 | 0.0 | 3.3 |  | 192 |
| *Parus montanus* | 0.0 | 0.0 | 0.0 | 0.0 | 3.8 | Yes | 303 |
| *Parus ater* | 0.0 | 0.0 | 0.0 | 0.8 | 9.0 | Yes | 603 |
| *Parus varius* | 0.0 | 0.0 | 0.5 | 1.7 | 10.0 | Yes | 791 |
| *Parus major* | 0.0 | 0.3 | 1.3 | 2.7 | 14.2 | Yes | 1049 |
| *Sitta europaea* | 0.0 | 0.0 | 0.0 | 0.0 | 10.0 | Yes | 324 |
| *Certhia familiaris* | 0.0 | 0.0 | 0.0 | 0.0 | 1.2 |  | 59 |
| *Zosterops japonicus* | 0.0 | 0.0 | 0.5 | 2.8 | 28.7 | Yes | 833 |
| *Emberiza cioides* | 0.0 | 0.0 | 0.7 | 2.3 | 14.7 | Yes | 1028 |
| *Emberiza yessoensis* | 0.0 | 0.0 | 0.0 | 0.0 | 14.2 |  | 11 |
| *Emberiza fucata* | 0.0 | 0.0 | 0.0 | 0.0 | 9.0 | Yes | 143 |
| *Emberiza sulphurata* | 0.0 | 0.0 | 0.0 | 0.0 | 4.0 |  | 115 |
| *Emberiza spodocephala* | 0.0 | 0.0 | 0.0 | 0.0 | 24.8 | Yes | 440 |
| *Emberiza variabilis* | 0.0 | 0.0 | 0.0 | 0.0 | 1.7 |  | 109 |
| *Emberiza schoeniclus* | 0.0 | 0.0 | 0.0 | 0.0 | 1.3 |  | 51 |
| *Carduelis sinica* | 0.0 | 0.0 | 0.5 | 1.8 | 10.7 | Yes | 983 |
| *Pinicola enucleator* | 0.0 | 0.0 | 0.0 | 0.0 | 0.8 |  | 4 |
| *Uragus sibiricus* | 0.0 | 0.0 | 0.0 | 0.0 | 4.3 |  | 165 |
| *Pyrrhula pyrrhula* | 0.0 | 0.0 | 0.0 | 0.0 | 0.8 |  | 106 |
| *Eophona personata* | 0.0 | 0.0 | 0.0 | 0.3 | 7.3 | Yes | 552 |
| *Coccothraustes coccothraustes* | 0.0 | 0.0 | 0.0 | 0.0 | 3.0 |  | 115 |
| *Passer rutilans* | 0.0 | 0.0 | 0.0 | 0.0 | 2.8 |  | 176 |
| *Passer montanus* | 0.0 | 0.0 | 0.0 | 1.5 | 82.5 | Yes | 873 |
| *Sturnus philippensis* | 0.0 | 0.0 | 0.0 | 0.0 | 3.3 |  | 141 |
| *Sturnus cineraceus* | 0.0 | 0.0 | 0.0 | 0.0 | 27.7 | Yes | 512 |
| *Garrulus glandarius* | 0.0 | 0.0 | 0.0 | 0.5 | 3.2 | Yes | 714 |
| *Cyanopica cyana* | 0.0 | 0.0 | 0.0 | 0.0 | 1.5 |  | 100 |
| *Nucifraga caryocatactes* | 0.0 | 0.0 | 0.0 | 0.0 | 0.7 |  | 37 |
| *Corvus corone* | 0.0 | 0.0 | 0.2 | 0.8 | 10.5 | Yes | 916 |
| *Corvus macrorhynchos* | 0.0 | 0.0 | 0.5 | 1.3 | 11.2 | Yes | 1036 |

**Appendix S2.** Definitions of wide-ranging and narrow-ranging species.

**Figure S1**.Range size for 113 native bird species that reproduce in terrestrial habitats in Japan. The threshold of 400 grids was used to define wide- and narrow-ranging species in the main text while 200 and 100 grids were used for sensitivity analyses (See Appendix S3 for results.)

**Appendix S3.** Sensitivity analysis of the definitions of species groups: we here defined wide-ranging species as those present in more than 200 or 100 grid squares and the others as narrow-ranging species. Wide-ranging species richness always showed a hump-shaped response to forest cover, with the peak at 65% forest cover (Figures S2a, S2g). Narrow-ranging species richness with the definitions of 200 and 100 grids showed a hump-shaped response to forest cover with the peak at 30% forest cover (Figure S2d) and a U-shaped response to forest cover with the peak always at 0% forest cover (i.e., open habitats) respectively (Figure S2h). Both definitions showed that narrow-species richness tended to be higher than open-habitats than forests. Because no narrow-ranging species with the definition of 100 grids was observed in > 20 transects, we conducted species-level analyses only for the definition of 200 grids. For wide-ranging species with this definition, the three response types (open-habitat, mosaic-habitat, and forest species) were well mixed, and 25.5% were categorized as mosaic-habitat species (the highest abundance in heterogeneous landscapes) (Figures S2b, S2c). For narrow-ranging species, 16.7% were categorized as mosaic-habitat species (Figures S2e, S2h). These results showed that wide-ranging species contained more of generalist species than narrow-ranging species, as also shown with the definition of 400 girds (please see main text).

**Figure S2.** Relationship between (a, d, g, h) species richness or (b, e) abundance and proportion of forest cover at a landscape scale, and (c, f) pie charts showing the proportions of species in the four categories of response type (criteria for categorization are shown in the text) for (a–c) wide-ranging and (d–f) narrow-ranging species (200 grids as a threshold) and (g) wide-ranging and (h) narrow-ranging species (100 grids as a threshold). Regression lines were based on the coefficients estimated with the best simultaneous autoregressive model using mean values other than the proportion of forest cover.

**Appendix S4.** Relationships among three indices of landscape heterogeneity.

**Figure S3.** Relationships between (a, d, g, j) Simpson’s diversity index and the proportion of forest cover, (b, e, h, k), edge density and Simpson’s diversity index and (c, f, i, l) edge density and the proportion of forest cover, at (a–c) 1-km, (d–f) 3-km, (g–i) 5-km and (j–l) 10-km scales. Regression lines were based on the coefficients estimated with generalized linear models using both linear and quadratic terms.

**Appendix S5.** Akaike Information Criterion (AIC) values of the two best models, which include (1) the proportion of forest cover as landscape heterogeneity (FRT) and (2) the range of elevation (RELV). For each species group, the model with the lower AIC value is shown in bold.

|  | AIC | | *Δ*AIC | |
| --- | --- | --- | --- | --- |
| Species richness | FRT | RELV | FRT | RELV |
| Total species | 14.9 | **14.1** | 0.8 | 0.0 |
| Wide-ranging species (>100 grids) | **26.1** | 27.6 | 0.0 | 1.5 |
| Wide-ranging species (>200 grids) | 41.5 | **40.4** | 1.0 | 0.0 |
| Wide-ranging species (>400 grids) | **49.2** | 52.3 | 0.0 | 3.1 |
| Narrow-ranging species (>100 grids) | **364.7** | 371.4 | 0.0 | 6.7 |
| Narrow-ranging species (>200 grids) | **649.0** | 649.9 | 0.0 | 0.9 |
| Narrow-ranging species (>400 grids) | **666.7** | 671.7 | 0.0 | 4.9 |

**Appendix S6.** Akaike Information Criterion (AIC) values of the seven models with different set of landscape variables: (1) the proportion of forest cover (FRT) and its quadratic term (FRT^2^), (2) Simpson’s diversity index added (SDI), (3) Edge density (EGD), (4) FRT and SDI, (5) FRT and EDG, (6) FRT, FRT^2^ and SDI, and (7) FRT, FRT^2^ and EDG. Note that SDI and EDG were not used simultaneously in one model to avoid multicollinearity. For each species group, the model with the lowest AIC value is shown in bold. Results showed that FRT and FRT^2^ were more important variables than SDI and EDG regardless of combinations of landscape variables (except for narrow-ranging species defined by 200 girds).

|  | AIC | | | | | | | *Δ*AIC | | | | | | |
| --- | --- | --- | --- | --- | --- | --- | --- | --- | --- | --- | --- | --- | --- | --- |
| Species richness | 1 | 2 | 3 | 4 | 5 | 6 | 7 | 1 | 2 | 3 | 4 | 5 | 6 | 7 |
| Total species | **15.6** | 20.8 | 21.2 | 17.1 | 17.7 | 17.8 | 17.4 | 0.0 | 5.2 | 5.6 | 1.5 | 2.1 | 2.2 | 1.8 |
| Wide-ranging species (>100 grids) | **27.9** | 35.7 | 35.6 | 34.1 | 32.9 | 29.7 | 29.4 | 0.0 | 7.8 | 7.7 | 6.2 | 5.0 | 1.8 | 1.5 |
| Wide-ranging species (>200 grids) | **42.6** | 51.5 | 50.6 | 48.0 | 46.0 | 44.5 | 43.4 | 0.0 | 8.9 | 8.0 | 5.4 | 3.4 | 1.9 | 0.8 |
| Wide-ranging species (>400 grids) | **49.6** | 65.7 | 65.5 | 58.0 | 57.8 | 51.5 | 51.4 | 0.0 | 16.1 | 15.9 | 8.4 | 8.2 | 1.9 | 1.8 |
| Narrow-ranging species (>100 grids) | **364.7** | 383.8 | 385.6 | 368.9 | 371.8 | 365.4 | 365.5 | 0.0 | 19.1 | 20.9 | 4.2 | 7.1 | 0.7 | 0.8 |
| Narrow-ranging species (>200 grids) | 658.8 | 659.4 | 661.3 | 653.7 | 659.3 | **651.5** | 660.8 | 7.3 | 7.9 | 9.8 | 2.2 | 7.8 | 0.0 | 9.3 |
| Narrow-ranging species (>400 grids) | **668.3** | 674.7 | 676.0 | 673.8 | 675.7 | 670.3 | 670.1 | 0.0 | 6.4 | 7.7 | 5.5 | 7.4 | 2.0 | 1.8 |

**Appendix S7.** Relationships between species richness and environmental factors.

**Figure S4.** Relationships between species richness and (a–c) number of surveys, (d–f) mean annual temperature, (g–i) annual precipitation, (j–l) actual evapotranspiration, (m–o) proportion of forest cover at a local scale, and (p–r) Simpson’s diversity index for (a, d, g, j, m, p) total species, (b, e, h, k, n, q) wide-ranging species, and (c, f, i, l, o, r) narrow-ranging species. Regression lines are based on the coefficients estimated in the best simultaneous autoregressive model using mean values other than the focal variable.

**Appendix S8.** *z* values (estimates/standard errors) of linear (L) and quadratic (Q) terms of each variable in the best simultaneous autoregressive models for the abundance of 57 species. A blank space means that the variable is not included in the best model. Abbreviations are the same as Table 1.

| Scientific name | Inter– | AMT | | APP | | AET | | FRT_local_ | | FRT | | SDI | Response^*^ | | |
| --- | --- | --- | --- | --- | --- | --- | --- | --- | --- | --- | --- | --- | --- | --- | --- |
|  | cept | L | Q | L | Q | L | Q | L | Q | L | Q | L | Scale | FRT_local_ | FRT |
| *Milvus Migrans* | –7.8 | –1.8 | –2.5 | –2.3 | 1.9 | 2.8 |  | –3.6 |  |  |  | 1.5 | 3 | O | N |
| *Butastur indicus* | –53.3 | 2.5 |  |  |  |  |  |  |  |  | –1.8 |  | 1 | N | M |
| *Syrmaticus soemmerringii* | –84.6 |  |  |  | 3.7 |  |  |  |  | 2.6 |  |  | 1 | N | F |
| *Phasianus colchicus* | –11.3 |  |  |  | –1.7 |  | –2.4 | –2.8 | 3.3 | –3.2 | –2.7 |  | 3 | O | M |
| *Streptopelia orientalis* | 3.3 | 3.1 |  |  |  | –3.5 |  | –4.8 | –3.5 | –4.8 |  |  | 10 | M | O |
| *Sphenurus sieboldii* | –11.1 | –2.7 | –3.4 |  |  | 2.2 | 3.3 |  | –1.4 |  |  |  | 1 | M | N |
| *Cuculus fugax* | –58.2 | –4.0 | –2.6 | 3.6 |  | 2.7 |  |  |  | 2.1 | 2.4 |  | 10 | N | F |
| *Cuculus canorus* | –14.8 | –5.6 | –2.6 |  |  | 1.8 |  | –1.7 |  | –4.4 |  | –1.5 | 1 | O | O |
| *Cuculus saturatus* | –16.2 | –3.7 | –3.6 |  |  |  | 4.3 |  |  | 3.4 |  |  | 3 | N | F |
| *Cuculus poliocephalus* | 0.2 |  | –2.9 |  | –1.7 | 2.5 |  | –1.6 | –2.9 |  | –2.9 | –2.7 | 1 | M | M |
| *Halcyon coromanda* | –24.3 |  |  | 2.1 | 1.6 |  |  |  |  | 2.8 | 2.2 |  | 1 | N | F |
| *Picus awokera* | –11.3 | 3.6 |  |  |  |  |  | 2.5 |  | 3.0 |  |  | 1 | F | F |
| *Dendrocopos major* | –11.4 | –8.1 | –4.5 |  |  |  | 1.8 |  | –3.8 |  | 1.6 | 3.1 | 3 | M | O |
| *Dendrocopos leucotos* | –47.6 |  | –1.6 |  |  | –1.7 |  |  |  | 3.1 | 1.9 | –1.7 | 1 | N | F |
| *Dendrocopos kizuki* | 6.1 | 8.2 |  |  |  |  |  |  | –8.1 |  |  | 3.0 | 3 | M | N |
| *Alauda arvensis* | –12.7 |  |  |  |  | –1.8 | –2.3 |  | 8.9 | –5.4 |  | –4.1 | 1 | O | O |
| *Hirundo rustica* | –1.8 | 5.1 | 3.3 |  |  | –2.6 |  | –6.3 |  | –2.6 |  | 2.0 | 3 | O | O |
| *Delichon urbica* | –18.8 |  | –1.5 |  | –2.1 |  | 2.5 | –3.5 |  | 3.9 |  |  | 10 | O | F |
| *Motacilla cinerea* | –13.3 | –2.8 | –1.5 |  |  | 2.6 |  |  |  | 4.5 | 1.5 | 1.9 | 3 | N | F |
| *Motacilla alba* | –18.0 |  |  |  |  | –1.7 |  | –3.4 | –3.3 | –3.1 |  |  | 1 | M | O |
| *Motacilla grandis* | –18.0 | 2.4 |  |  |  |  |  | –5.8 |  |  | –4.0 |  | 1 | O | M |
| *Pericrocotus divaricatus* | –10.0 | 4.2 |  |  |  | –1.6 |  | 2.3 |  | 1.8 |  |  | 3 | F | F |
| *Hypsipetes amaurotis* | 19.4 | 11.9 | –2.7 | –3.8 |  |  |  | –2.8 | –7.0 | 3.1 |  | 3.0 | 1 | M | F |
| *Lanius bucephalus* | –11.6 | –3.0 | –2.7 |  |  |  |  | –8.5 |  |  |  |  | 3 | O | N |
| *Troglodytes troglodytes* | –3.5 | –8.1 | –2.7 | 4.5 |  | 2.5 |  | 3.2 |  | –1.9 |  | –3.4 | 5 | F | O |
| *Luscinia cyane* | –11.0 | –7.5 |  |  | –2.3 |  |  | 4.1 |  |  | 2.1 |  | 1 | F | O |
| *Zoothera dauma* | –33.7 |  | –2.1 |  |  |  |  |  |  | 5.0 | 2.4 |  | 1 | N | F |
| *Turdus cardis* | –5.3 | –4.0 | –5.8 |  | –1.9 | 3.7 |  |  |  | 5.1 |  |  | 1 | N | F |
| *Turdus chrysolaus* | –30.4 | –6.4 |  |  |  |  |  |  |  |  | 2.3 |  | 10 | N | O |
| *Urosphena squameiceps* | –3.0 | 3.9 | –2.1 | –2.7 |  |  |  | 4.3 | 2.3 | 3.3 |  |  | 1 | F | F |
| *Cettia diphone* | 15.0 | 3.3 |  | –2.0 |  |  |  | –2.8 | –3.7 |  | –3.9 | –3.2 | 3 | M | M |
| *Acrocephalus bistrigiceps* | –16.5 | –4.2 | –1.8 |  |  |  |  |  | 6.2 | –3.4 |  |  | 3 | O | O |
| *Acrocephalus arundinaceus* | –15.0 |  | –1.8 |  |  |  |  |  | 8.3 | –1.9 | 4.4 |  | 1 | O | O |
| *Phylloscopus borealis* | –14.4 | –3.5 | 4.2 |  | 2.4 |  |  | 3.7 |  | –4.1 |  |  | 10 | F | O |
| *Phylloscopus coronatus* | –1.4 |  | –5.8 |  | –3.6 | –2.7 | 4.1 | 4.8 |  |  | 1.4 |  | 10 | F | O |
| *Cisticola juncidis* | –17.1 |  |  |  |  |  |  |  | 6.4 | –3.1 |  | –3.6 | 1 | O | O |
| *Ficedula narcissina* | 2.9 |  | –3.3 |  |  |  |  | 8.5 |  | –3.4 | –2.7 |  | 3 | F | M |
| *Cyanoptila cyanomelana* | –2.8 | –1.8 | –4.1 | 2.2 | –2.0 |  | 2.4 | 2.8 |  | 4.7 | 2.4 |  | 1 | F | F |
| *Muscicapa dauurica* | –32.6 |  |  |  |  | –3.1 |  | 1.5 | –1.5 | –4.5 |  | –2.9 | 3 | F | O |
| *Terpsiphone atrocaudata* | –17.5 | 4.5 |  |  |  |  |  | 3.5 | 1.6 |  | –2.4 |  | 5 | F | M |
| *Aegithalos caudatus* | 1.6 | 5.8 |  |  |  |  |  | 5.1 |  | –1.9 | –2.4 |  | 1 | F | M |
| *Parus montanus* | –12.5 | –4.2 |  |  |  | 1.5 |  | 1.7 |  | 3.8 | 3.2 | 1.6 | 3 | F | F |
| *Parus ater* | –4.5 | –7.0 | –3.6 |  | 2.0 | 3.6 |  | 3.4 |  | 5.4 | 3.6 |  | 3 | F | F |
| *Parus varius* | 6.0 | 9.2 |  |  |  |  |  | 4.0 | –2.0 |  | –4.1 | –1.6 | 10 | F | M |
| *Parus major* | 8.3 | 2.8 | –3.1 | –1.5 |  |  |  | 1.7 | –5.2 |  | 2.8 | 1.6 | 10 | F | O |
| *Sitta europaea* | –15.2 | –4.5 | –1.5 |  | 4.0 |  | 1.9 | 2.4 |  |  |  | –2.8 | 1 | F | N |
| *Zosterops japonicus* | 4.2 | 7.9 | 2.3 | –3.6 | 2.2 | 1.5 |  | 1.5 | –1.8 |  | –4.1 | 1.5 | 1 | F | M |
| *Emberiza cioides* | 9.4 | 4.4 | –2.6 |  | –1.9 |  |  | –5.5 | –3.5 |  | –3.1 |  | 1 | M | M |
| *Emberiza fucata* | –13.0 | –3.3 |  | 2.3 |  |  |  | –1.9 | 6.9 | –1.9 | –5.3 |  | 5 | O | M |
| *Emberiza spodocephala* | –1.2 | –3.1 | –3.1 |  |  |  | 1.9 |  |  | –4.7 | –2.1 |  | 3 | N | O |
| *Carduelis sinica* | 4.7 | 2.8 | –1.5 | –5.4 | 2.4 |  |  | –3.9 | –3.8 | –3.7 | –3.3 |  | 1 | M | M |
| *Eophona personata* | –3.5 |  | –4.0 |  |  |  |  | 3.3 |  |  | –3.5 |  | 10 | F | M |
| *Passer montanus* | –0.1 | 5.6 | 2.4 |  | –1.8 |  |  | –6.7 | –1.7 | –3.6 | 2.1 |  | 5 | O | O |
| *Sturnus cineraceus* | –8.9 |  |  |  |  |  |  | –4.8 |  | –1.8 | 4.7 | 2.3 | 3 | O | O |
| *Garrulus glandarius* | –4.1 | –3.2 | –5.7 | 3.6 | –2.3 |  |  |  | –1.7 | 5.7 | 3.7 |  | 1 | M | F |
| *Corvus corone* | 0.9 | 3.4 |  | –2.0 |  |  |  | –5.0 | –3.2 | –3.6 |  |  | 3 | M | O |
| *Corvus macrorhynchos* | 5.7 | 4.6 |  | –1.8 |  |  |  |  | –5.2 | –5.3 |  |  | 1 | M | O |

^*^For each species, under “Response”, “Scale” indicates the best spatial scale (km) of landscape variables (blank indicates that landscape variable is not included in the best model), and “FRT_local_” and “FRT” indicate the type of response to forest cover at the local and best landscape scale, respectively: (O) Open-habitat species, (M) Mosaic-habitat species, (F) Forest species and (G) No response (see the main text for the definition).

**Appendix S9.** Akaike Information Criterion (AIC) values of the best and interaction models, in which an interaction term between mean annual temperature and proportion of forest cover was added into the best model. For each species group, the model with the lowest AIC value is shown in bold.

|  | AIC | | *Δ*AIC | |
| --- | --- | --- | --- | --- |
| Species richness | Best | Interaction | Best | Interaction |
| Total species | **14.9** | 18.5 | 0.0 | 3.6 |
| Wide-ranging species (>100 grids) | **26.1** | 29.7 | 0.0 | 3.6 |
| Wide-ranging species (>200 grids) | **41.5** | 45.0 | 0.0 | 3.6 |
| Wide-ranging species (>400 grids) | **49.2** | 52.9 | 0.0 | 3.7 |
| Narrow-ranging species (>100 grids) | 364.7 | **348.6** | 16.1 | 0.0 |
| Narrow-ranging species (>200 grids) | 651.5 | **646.1** | 5.4 | 0.0 |
| Narrow-ranging species (>400 grids) | 666.7 | **666.4** | 0.3 | 0.0 |
